# Defining Alcohol-Related Phenotypes in Humans: The Collaborative Study on the Genetics of Alcoholism

**Published:** 2002

**Authors:** Laura Jean Bierut, Nancy L. Saccone, John P. Rice, Alison Goate, Tatiana Foroud, Howard Edenberg, Laura Almasy, P. Michael Conneally, Raymond Crowe, Victor Hesselbrock, T. K. Li, John Nurnberger, Bernice Porjesz, Marc A. Schuckit, Jay Tischfield, Henri Begleiter, Theodore Reich

**Affiliations:** Laura Jean Bierut, M.D., is an associate professor, Nancy L. Saccone, Ph.D, is an assistant professor, John P. Rice, Ph.D., is a professor, Alison Goate, D.Phil., is a professor, and Theodore Reich, M.D., is the Samuel and Mae S. Ludwig Professor, all at Washington University School of Medicine, St. Louis, Missouri. Tatiana Foroud, Ph.D., is an associate professor, Howard Edenberg, Ph.D., is a Chancellor’s Professor, P. Michael Conneally, PhD., is a distinguished professor, T. K. Li, M.D., is a distinguished professor, and John Nurnberger, Jr., M.D., Ph.D., is the Joyce and Iver Small Professor of Psychiatry, all at Indiana University School of Medicine, Indianapolis, Indiana. Laura Almasy, Ph.D., is an associate scientist at the Southwest Foundation, San Antonio, Texas. Raymond Crowe, M.D., is the George Winokur Professor at the University of Iowa, Iowa City, Iowa. Victor Hesselbrock, Ph.D., is a professor at the University of Connecticut Health Center, Farmington, Connecticut. Bernice Porjesz, Ph.D., and Henri Begleiter, M.D., are professors at the State University of New York, Health Sciences Center at Brooklyn, New York. Marc A. Schuckit, M.D., is a professor at the University of California–San Diego, San Diego, California. Jay Tischfield, Ph.D., is a professor at Rutgers University, Piscataway, New Jersey

**Keywords:** genetic theory of AODU (alcohol and other drug use), AODR (alcohol and other drug related) genetic markers, phenotype, chromosome, AOD dependence potential, genetic variance, genetic trait, comorbidity, major depression, AOD intake per occasion, electroencephalography, genetic linkage, genetic correlation analysis

## Abstract

Alcoholism is a disease that runs in families and results at least in part from genetic risk factors. The Collaborative Study on the Genetics of Alcoholism (COGA) is a Federally funded effort to identify and characterize those genetic factors. The study involves more than 1,000 alcoholic subjects and their families, with researchers conducting comprehensive psychological, physiological, electrophysiological, and genetic analyses of the participants. These analyses have identified several traits, or phenotypes, that appear to be genetically determined, such as the presence of alcohol dependence, the level of response to alcohol, the presence of coexisting depression, or the maximum number of drinks a person consumes per occasion. Genetic analyses have identified regions on several chromosomes that are associated with these phenotypes and need to be studied further.

Alcohol dependence is a common, complex disorder that clusters in families. Strong evidence from twin and adoption studies suggests that alcoholism is in part caused by a genetic predisposition. (Definitions of the terms “alcoholism” or “alcohol dependence” are discussed later in this article.) Many other traits that are associated with the risk for alcoholism also cluster in families and have genetic underpinnings. These traits, or phenotypes, include a person’s response to alcohol; the maximum amount of alcohol a person consumes on a single occasion; and biological measurements, such as brain electrophysiological measures. Certain psychiatric disorders that commonly co-occur with and may increase the risk for alcoholism, such as depression, antisocial personality disorder, or abuse of other drugs, also may be caused partly by genetic factors. Genetic studies of complex disorders can use analyses of such correlated characteristics to improve the likelihood of finding genes that are associated with the development of these disorders. To use this strategy, researchers must conduct comprehensive assessments in multiple domains (e.g., behavioral responses and physiological reactions). This approach allows for the examination of multiple characteristics that may be influenced by the same underlying genes.

The Collaborative Study on the Genetics of Alcoholism (COGA) is an ambitious research effort funded by the National Institute on Alcohol Abuse and Alcoholism to elucidate the genetic factors contributing to the risk of alcoholism. The investigators participating in this multicenter study have performed genetic studies of alcohol dependence and several related phenotypes. This article describes the COGA study and the methods of genetic analyses used in it, presents some of the phenotypes that were assessed, and includes a brief review of some of the findings regarding these phenotypes. More detailed information about recent findings of the COGA study is provided in the sidebar by Edenberg, pp. 214–218, in this issue.

## Design of the COGA Study

COGA is a large-scale effort to detect and map, or determine the location of, genes that influence susceptibility for developing alcohol dependence. To maximize the potential of success in this project, the study investigators used a strategy that involved systematic recruitment of a large number of alcohol-dependent patients and their relatives; comprehensive clinical assessment; and analysis of targeted biological traits, such as brain wave measurements.

As a first step, the COGA investigators recruited alcohol-dependent people from chemical dependency treatment centers. These patients (also called index cases, or probands) as well as their family members were invited to participate in the study. All participants were interviewed to assess various domains, including the presence of alcohol abuse and dependence; other psychiatric disorders (e.g., depression) and other medical illnesses; the participant’s family history of alcoholism; and other behaviors. Diagnoses of alcohol dependence and other psychiatric disorders were established using a structured, comprehensive, diagnostic interview called the Semi-Structured Assessment for the Genetics of Alcoholism (SSAGA), which was developed specifically for the COGA study (Bucholz et al. 1994; Hesselbrock et al. 1999). To be recruited into the COGA study, probands had to meet both the diagnostic criteria for alcohol dependence specified in the *Diagnostic and Statistical Manual of Mental Disorders, Third Edition, Revised* (DSM–III–R) (American Psychiatric Association [APA] 1987) and the criteria for definite alcoholism specified by Feighner and colleagues (1972). The recruitment procedures have been fully described by Begleiter and colleagues (1995).

Overall, the COGA investigators recruited more than 1,200 probands and their family members, resulting in interviews of more than 11,000 people. Among those participants, the investigators selected 262 severely affected families—families in which at least 3 interviewed first-degree relatives^1^ were diagnosed with alcohol dependence—for further intensive assessment for genetic studies. More-distant relatives in these severely affected families were also recruited into this phase of the study, so that approximately nine people per family participated in the genetic studies.

For the families recruited for the genetic analyses, the investigators gathered additional information from various domains. For example, they collected blood samples from a total of 2,282 people for further analysis, as described in the following section. Furthermore, the participants underwent additional biological measurements, including measurements of brain activity, such as electroencephalograms (EEGs) and event-related potentials (ERPs). EEGs measure fundamental electrical brain activity. ERPs are changes in the ongoing electrical brain activity that occur as a person responds to a stimulus. EEGs and ERPs can be measured reliably, and there is strong evidence that the patterns of these brain waves are genetically determined (Porjesz et al. 2002). Both measures allow important assessments of underlying biological features related to alcoholism. The participants also completed several questionnaires—for instance, the Self-Rating of the Effects of Alcohol (SRE) (Schuckit et al. 2001)—to determine their response to alcohol.

### Genetic Analysis

Because the researchers did not know in advance which chromosomes would carry genetic factors influencing the development of alcoholism and related phenotypes, the genetic analyses involved a survey of all chromosomes—that is, the entire genome. To follow the inheritance of different regions of each chromosome, the researchers used more than 300 microsatellite markers—short, repeated DNA sequences that exist in many variants (i.e., alleles) and whose locations on the chromosomes are known. Every person carries two copies of each marker, one inherited from the mother and one inherited from the father. Because of their diversity, these two copies of each marker are likely to differ, allowing researchers to follow the inheritance of the corresponding chro mosomal regions in a family. Such a genomewide search strategy allows for the discovery of novel genes not previ ously considered candidates for influ encing alcoholism risk.

The investigators then used statistical tests to search for relationships between the pattern of inheritance of the DNA markers and the traits under investigation (e.g., alcohol dependence) in the study families. If a relationship existed between certain genetic markers and a given phe notype—for example, if a variant of a marker occurred more commonly in family members with the phenotype than would be expected by chance—this served as evidence of genetic linkage.

The investigators used both qualita tive and quantitative analytic tech niques in their genetic analyses. Quali tative techniques are used to assess traits that can only be either present or absent (e.g., alcohol dependence). One approach chosen in the COGA study was the affected sibling pair method, which examines the degree to which sibling pairs, both of whom exhibit the phenotype under investigation, share genetic markers. This method looks for deviation from the assumption that siblings on average share 50 percent of their genes. Thus, two affected siblings would share genetic factors that contribute to a given phenotype more than 50 percent of the time. Phenotypes studied using this method included alcohol dependence, low level of response to alcohol, the presence of alcoholism or depression, and being unaffected by alcoholism (all of these phenotypes are described in the follow ing sections). The statistical power of these analyses was limited because they involved only study participants who exhibited the phenotype under investi gation and thus were unable to take advantage of the detailed assessment available for all study participants.

The second method of genetic anal ysis examined quantitative phenotypes—traits that are present to varying degrees (e.g., the maximum number of drinks a person has consumed per occasion). The advantage of quantitative methods is that assessments are available on affected as well as unaffected people; thus, these methods have greater statis tical power because they include more people. Phenotypes studied using quantitative methods included the severity scale of alcohol dependence, level of response to alcohol, maximum number of drinks per occasion, and electrophysiological (i.e., EEG and ERP) measurements.

## Phenotypes Investigated in the COGA Study

The comprehensive analyses included in the COGA study allowed for the analysis of numerous phenotypes, thereby increasing the likelihood that investigators will find relevant genes. For example, by choosing a phenotype that occurs only in the most severely affected people, researchers may reduce the generalizability of findings. Conversely, by focusing on a phenotype that is very common, researchers define an overly broad study population that may be highly variable (i.e., heterogeneous) with respect to genetic findings. The following sections summarize some of the phenotypes included in the COGA analyses. This discussion focuses on phenotypes that demonstrated linkage with DNA regions on chromosomes 1 and 4. Other analyses, however, also found linkage of various phenotypes with other chromosomes.

### “Alcohol Dependence” Phenotype

The phenotype of “alcohol dependence” was studied both as a categorical variable—that is, whether a person was affected by alcoholism according to the COGA criteria—and as a quantitative variable (i.e., according to severity). The severity of alcohol dependence was classified using multiple commonly used sets of diagnostic criteria:

- The DSM–III–R criteria
- The criteria specified in the fourth edition of the DSM (DSM–IV) (APA 1994)
- The criteria specified in the World Health Organization’s *International Classification of Diseases and Related Health Problems, Tenth Revision* (ICD–10) (WHO 1992–1994)
- The COGA criteria as described above (Reich et al. 1998).

These different definitions of alcoholism represent various grades of severity of the disorder, with DSM–III–R criteria the least stringent and the ICD–10 criteria the most stringent. Consequently, the DSM–III–R criteria identify the largest group of people affected with alcoholism, and the ICD–10 criteria identify the smallest, most severely affected group.

When the COGA investigators compared genetic markers among sibling pairs in which both siblings met the COGA or ICD–10 criteria for alcohol dependence, they identified one DNA region (i.e., a locus) on chromosome 1 that showed genetic linkage with the “alcohol dependence” phenotype (Reich et al. 1998; Foroud et al. 2000). Moreover, when the researchers analyzed alcoholism as an underlying quantitative trait, they found evidence for genetic linkage with a region on chromosome 4 (Williams et al. 1999).

### “Low Level of Response” Phenotype

One characteristic related to the development of alcohol dependence is the level of subjective response to alcohol (Schuckit et al. 2001). People who are at high risk of developing alcoholism (e.g., children of alcoholics) more frequently report that they need to consume greater amounts of alcohol to feel alcohol’s effects than do other people. These people are said to have a low level of response to alcohol; this characteristic is a powerful predictor of the subsequent development of alcohol dependence, and twin studies have demonstrated that this trait is genetically influenced.

The COGA investigators assessed the participants’ response to alcohol using the SRE questionnaire. SRE scores can be used either categorically or quantitatively. For categorical analyses, a certain score on the SRE (e.g., the bottom third of the scores) is designated as a threshold, and people scoring below this value are classified as having a low response to alcohol. For quantitative measurements, the scores of all participants are considered and ordered along a continuum from lowest score (i.e., lowest response to alcohol) to highest score (i.e., highest response).

Qualitative assessments using the affected sibling design found evidence for genetic linkage of the “low level of response” phenotype with the same region on chromosome 1 that was linked with the “alcohol dependence” phenotype (Schuckit et al. 2001). Quantitative measures also found some evidence of linkage; however, although suggestive, these results were not statistically significant.^2^

### “Alcoholism or Depression” Phenotype

Many alcohol-dependent people also suffer from major depressive disorder (Nurnberger et al. 2001), and twin studies suggest that both disorders share some common genetic factors. Consistent with previous findings, comorbid alcoholism and depression also commonly occurred in the COGA families. Given the potential genetic relationship between alcohol dependence and major depressive disorder, the COGA investigators defined a composite phenotype termed “alcoholism or depression.” Study participants who met criteria for alcohol dependence, depression, or both disorders were considered affected with this phenotype. Depression was defined as the presence of a lifetime history of major depressive disorder or depressive syndrome as specified in the DSM–III–R. A depressive syndrome is diagnosed in people who meet the diagnostic criteria for major depressive disorder but in whom the depression may have been precipitated by heavy alcohol or other drug use, medications, or other medical illnesses.

The “alcoholism or depression” phenotype was studied as a qualitative trait using the affected sibling design—that is, the analysis included sibling pairs in which both siblings were alcohol dependent and/or suffered from depression. Accordingly, in contrast to the other sibling analyses reported here, genetic analyses using this composite phenotype included people who were afflicted with depression but were not alcohol dependent. Because depression was more common among female than among male study participants, this analysis resulted in the inclusion of more sibling pairs with one or two affected sisters.

The genetic analyses found evidence for genetic linkage between this phenotype and the chromosome 1 locus that was also linked to alcohol dependence and low level of response to alcohol (Nurnberger et al. 2001). In fact, of the traits analyzed, the “alcoholism or depression” phenotype showed the strongest association with that locus.^3^ The COGA findings regarding the association between alcoholism and depression are discussed in more detail in the article by Nurnberger and colleagues, pp. 233–240, in this issue.

### “Unaffected” Phenotype

The COGA investigators also defined a phenotype termed “unaffected,” which was assigned to people who used alcohol but did not meet the criteria for alcohol dependence (Reich et al. 1998).^4^ Analysis of people with the “unaffected” phenotype, who were relatively rare in the COGA families, is interesting because these people developed few or no alcohol-related problems despite living in a family environment characterized by the presence of several alcoholic family members and, consequently, excessive alcohol consumption. This suggests that these people may carry genetic factors that help protect against the development of alcoholism.

The genetic analyses of the COGA studies provided some evidence of a genetic linkage of the “unaffected” phenotype with the same region on chromosome 4 that had also been identified during the quantitative analyses of the “alcohol dependence” phenotype (Reich et al. 1998). This finding is intriguing because this DNA region is located near the genes for alcohol dehydrogenase, an enzyme involved in alcohol metabolism. Some variants of this enzyme have been shown to protect against the development of alcoholism in Asian populations (Higuchi et al. 1995, 1996) but are less commonly found in Caucasian populations.

### “Maximum Number of Drinks” Phenotype

Another alcoholism-related trait assessed in the COGA study was the maximum number of drinks a person ever consumed in a 24-hour period, which was determined based on the participants’ response to the question, “What is the largest number of drinks you have ever had in a 24-hour period?” (Saccone et al. 2000). The examination of this variable was motivated by twin studies that demonstrated a moderate genetic influence on the maximum number of drinks a person consumes in 24 hours. The COGA investigators found a significant association in the study participants between the maximum number of drinks consumed and the risk of developing alcoholism. Thus, none of the participants who reported drinking a maximum of 2 drinks within a 24-hour period exhibited any symptoms of problem drinking. However, the rate of alcoholism increased with increasing maximum amounts of alcohol consumption in a 24-hour period. For instance, among participants who reported drinking 9 or more drinks in a 24-hour period, 65 percent of men and 53 percent of women were diagnosed with alcohol dependence.

The “maximum number of drinks” phenotype was a quantitative measurement; accordingly, the genetic analyses included both alcoholic and nonalcoholic people. These analyses found a genetic linkage between this phenotype and the locus on chromosome 4 that had already shown linkage with the “unaffected” phenotype and with severity of alcoholism (Saccone et al. 2000).

### Electrophysiological Phenotypes

Several brain electrophysiological measures, such as EEGs and ERPs, are altered in people with various psychiatric disorders, including alcoholism (Porjesz and Begleiter 1998). For example, an ERP brain wave called P300 frequently is smaller in size (i.e., has a lower amplitude) in alcoholics than in nonalcoholic people. Increasing evidence also suggests that the variations in brain electrophysiological activity predate the development of alcoholism. For example, compared with people without a family history of alcoholism, alterations in EEGs and ERPs exist both in alcohol-dependent people and in people who are not alcoholic but are at risk for alcoholism because they are relatives of alcoholics. These electrophysiological measures therefore represent biological markers that are related to a predisposition for developing alcoholism. Accordingly, EEGs and ERPs were included in the COGA analyses, particularly measurements of the P300 brain wave and another ERP brain wave called N400.

The genetic analyses found that several regions on chromosome 4 were linked to the electrophysiological phenotypes:

- The DNA region that previously was found to be linked with the “unaffected” and “maximum number of drinks” phenotypes also showed some genetic linkage in a combined analysis of alcohol dependence and P300 amplitude (Williams et al. 1999).
- A second region on chromosome 4 showed evidence of linkage to the amplitudes of the P300 and N400 waves when the investigators used a different stimulus to elicit the ERPs (Almasy et al. 2001). This same region appeared to be linked to alterations in certain EEG measures (Porjesz et al. 2002).
- A third area on chromosome 4 showed strong evidence of linkage to changes in another EEG measurement (Porjesz et al. 2002). This region is of interest because it contains genes for proteins (i.e., receptors) that interact with a brain chemical called gamma-aminobutyric acid (GABA), which is responsible for important aspects of nerve cell activity and influences EEG activity. Further genetic analyses indicated that a variant of a GABA receptor gene in this area is associated with changes in EEG activity.

## Summary

In the COGA study, researchers use comprehensive measurements of numerous behavioral and biological traits associated with alcoholism to conduct a thorough genetic analysis of this complex disorder. These analyses have identified several chromosomal regions, particularly on chromosomes 1 and 4, that appear to be linked to several alcohol-related phenotypes and that are now the targets of more detailed analyses. The fact that some of these areas appear to be linked with more than one phenotype strengthens the evidence that genetic factors in these chromosomal regions contribute to the development of alcoholism.

In the study of complex diseases such as alcoholism, it is important to perform a complete assessment of the disorder and its related characteristics. In COGA, there is a close relationship between the phenotypes studied and the genetic findings. This means that as the phenotypes of alcoholism and correlated characteristics are modified, linkage to different chromosomal regions is found. Although alcohol dependence is correlated with many behavioral features and physiological measurements, genetic linkage analyses of the traits studied have pointed to different genetic regions related to these traits. For example, three phenotypes—“alcohol dependence,” “low level of response” to alcohol, and “alcoholism or depression”—exhibit genetic linkage with the same area on chromosome 1, although each of these phenotypes was found in a different subset of participants. This finding suggests that a genetic factor may be located in the region that contributes to the development of multiple characteristics, such as alcohol dependence, depression, and a person’s initial response to alcohol, with the specific effect depending on other accompanying factors. Several other phenotypes—“unaffected,” “maximum number of drinks,” alcoholism severity, and the combined analysis of alcohol dependence and the P300 component of the ERP—show genetic linkage with a common region on chromosome 4 that is near the genes for alcohol dehydrogenase.

The evaluation of multiple traits in the COGA study has provided insights into various alcohol-related behaviors and biological characteristics that are associated with different underlying genetic factors. Future work in understanding the genetics of alcoholism requires a continued, thorough assessment of alcohol-related problems and correlated characteristics. In addition, researchers will have to make progress in the development of statistical analytic tools and molecular genetics to allow the study of more complex phenotypes and genetic information.

## Footnotes

1 First-degree relatives of a proband are his or her parents, siblings, and children.

2 The analyses were not corrected for the participants’ gender, weight, body mass index, and similar variables.

3 This variation in the strength of the association resulted in part from the different sample sizes used in the different analyses.

4 It is important to note that to be evaluated for the “unaffected” phenotype, study participants had to be current drinkers; abstinent participants were not included in the analysis of this phenotype.
